# Nitrogen Fertilizer and Straw Applications Affect Uptake of ^13^C,^15^N-Glycine by Soil Microorganisms in Wheat Growth Stages

**DOI:** 10.1371/journal.pone.0169016

**Published:** 2017-01-03

**Authors:** Lijie Yang, Lili Zhang, Chunxiao Yu, Dongpo Li, Ping Gong, Yan Xue, Yuchao Song, Yalan Cui, Timothy A. Doane, Zhijie Wu

**Affiliations:** 1 National Nutrition and Engineering Lab, Institute of Applied Ecology, Chinese Academy of Sciences, Shenyang Liaoning, China; 2 Graduate School, University of Chinese Academy of Sciences, Beijing, China; 3 Department of Land, Air, and Water Resources, University of California Davis, Davis California, United States of America; University of Oklahoma, UNITED STATES

## Abstract

This study investigated the influence of nitrogen (N) fertilizer and straw on intact amino acid N uptake by soil microorganisms and the relationship between amino acid turnover and soil properties during the wheat growing season. A wheat pot experiment was carried out with three treatments: control (CK), N fertilizer (NF) and N fertilizer plus rice straw (NS). We used stable isotope compound-specific analysis to determine the uptake of ^13^C,^15^N-glycine by soil microorganisms. In the NF treatment, microbial ^13^C,^15^N-glycine uptake was lower compared with CK, suggesting that inorganic N was the preferred N source for soil microorganisms. However, The application of straw with N fertilizer (in NS treatment) increased microbial ^13^C,^15^N-glycine uptake even with the same amount of N fertilizer application. In this treatment, enzyme activities, soil microbial biomass C and microbial biomass N increased simultaneously because more C was available. Soil mineral N and plant N contents all decreased substantially. The increased uptake of intact ^13^C,^15^N-glycine in the NS treatment can be attributed to direct assimilation by soil microorganisms to satisfy the demand for N when inorganic N was consumed.

## Introduction

Amino acids represent an important component of soil organic nitrogen (N) pools, accounting for 20–50% of the identifiable soil N pool upon acid hydrolysis[1, 2]. Studies with isotopic tracers have indicated that the half-life of the amino acid pool in soil solution ranges from 0.8 h to 5.9 h [3, 4]. Due to the rapid turnover of the free amino acid pool, they have been considered as a potentially important source of N for soil microorganisms and plants [5–7]. Soil microorganisms can assimilate amino acids directly or as NH_4_^+^ produced by ammonification [5, 8, 9]. If the N is not needed for metabolism, directly assimilated amino acids can be deaminated intracellularly and the surplus N may then be excreted into soil solution. Alternatively, the N may be incorporated into new amino acids, peptides or proteins [10]. If direct uptake of amino acids predominates in soil microorganisms, microbes can satisfy their N requirement with these organic N compounds, which may then make more inorganic N available to plants [11].

The direct uptake of amino acids by soil microorganisms may be important in soil ecosystems [3, 5, 12]. By using ^15^N isotope dilution and enrichment techniques, Barraclough (1997) demonstrated direct uptake of leucine and glycine. Geisseler et al. (2009) came to a similar conclusion, in that 55 to 62% of added amino acid-N was absorbed by the direct route when two types of wheat residues were added to soil samples. In this case, however, the ^15^N mirror image technique provided only indirect evidence for the assimilation of intact amino acids, as it only quantifies extracellular deamination of amino acids. By using ^13^C,^15^N-glycine addition, determination of the ^13^C and ^15^N enrichment of microbial biomass may show a significant linear correlation between^13^C and ^15^N enrichment, which indicates microbial uptake of intact glycine [13]. However, this method does not discern whether deamination occurred inside or outside the cell. By using compound-specific stable isotope analysis, it is possible to determine the fate of applied amino acids in soil and the uptake of intact amino acids by soil microorganisms [5, 14, 15].

Nitrogen fertilizers are critical in order to maintain crop production in agro-ecosystems [16, 17]. Over-application of N fertilizer, however, leads to serious side effects, such as soil quality deterioration and water contamination [18, 19]. In recent years, increasing attention has been given to plant residues as amendments in order to improve biological and chemical soil properties, as well as to reduce dependence on mineral fertilizers [20–22]. Plant residues can release amino acids and easily degradable proteins from lysed cells in fresh plant residues or via depolymerization of proteins in older vegetable tissue [23]. Furthermore, decomposition of plant residue in soil generates different compounds of C and N [24], which in turn may influence the relative significance of amino acid uptake. Therefore, the addition of plant residues may affect the uptake of amino acids by soil microorganisms. For example, in an aerobic incubation experiment, the addition of straw significantly increased the direct uptake of amino acids compared to a treatment without straw application [25].

Enzyme activities are directly involved in the transformation of complex C compounds to readily available nutrients for microbes and plants. For example, protease catalyzes the hydrolysis of protein to smaller, membrane-permeable peptides and amino acids [26]. Proteolysis is an important process in many ecosystems with regard to N cycling because it is considered a rate-limiting step during N mineralization [27]. β-D-glucosidase and cellobiohydrolase primarily degrade cellulose, and N-acetyl-β-D-glucominidase degrades chitin into soluble subunits that can be taken up by soil microorganisms [28]. Therefore, enzyme activity is an important part of our understanding of the interactions between C and N availability during the decomposition of plant residues.

Plant roots release large amounts of easily available C into the rhizosphere [29, 30]. The microorganisms stimulated by available C begin capturing N earlier than the roots, and under such circumstances available soil N is therefore largely captured by soil microorganisms. Soil microorganisms absorb available N earlier than roots due to it earlier stimulation of activity by available C and consequent earlier capture of N. With the rapid growth of microorganisms, available C in soil becomes depleted and more C inputs are required to sustain activity. Uptake of available C by soil microorganisms corresponds with the N which is immobilized in microbial biomass after being released into the soil [31]. This sequence results in changes in the availability of N for plants. Previous research has led to the conclusion that only inorganic N can be assimilated by soil microorganisms during these processes. It is now known that not only inorganic N, but also intact organic N, can be used directly to satisfy the need for N. Furthermore, differences in quality as well as quantity of root exudates may influence the uptake of intact organic N. Relatively little information is currently available about how much intact organic N can be used under conditions where growing plants are present.

The present study provides information about the influence of inorganic N alone and N plus plant residue on amino acid N utilization by soil microorganisms under controlled conditions with actively growing plants. We hypothesized that (1) amendment of soil with mineral N fertilizer alone would decrease the uptake of intact amino acids by soil microorganisms because inorganic N is the preferred source for soil microorganisms to proliferate; (2) uptake of intact amino acids would increase with the addition of N fertilizer and straw due to strong demand for N under the increased availability of C.

## Materials and Methods

The experiment was conducted at the National Field Observation and Research Station of Shenyang Agroecosystems (41°32′ N, 123°23′ E), which is located in Shengyang, Liaoning Province, China. The authority of the field is the Institute of Applied Ecology, Shenyang, which issued permission to use the field for the research work.

### Field site and soil sampling

The pot experiment was conducted under outdoor conditions in a wire house (with a roof of iron net) at the National Field Observation and Research Station of Shenyang Agroecosystems (41°32′ N, 123°23′ E) in 2014. Mean annual temperature and mean annual precipitation at the station are 7–8°C and 700 mm, respectively. Soil was taken from the top 20 cm of soil under continuous maize cultivation. The soil is classified as a Lixisol and had the following characteristics:19% sand; 63% silt; 18% clay [32], as well as a water holding capacity of 35%, a pH of 6.01, exchangeable NH_4_^+^-N of 16.6 mg kg^-1^, and NO_3_^-^-N of 26.4 mg kg^-1^. Total organic C and total N were 11.8 g C kg^-1^ dry soil and 1.22 g N kg^-1^dry soil.

### Experimental design

Soil was sieved (< 2 mm) and put into plastic pots (pot height: 20 cm; diameter: 18 cm). The amount of soil per pot was equivalent to 2 kg dry soil. Water was added to bring the soil to 0.18 g water g^-1^ soil (corresponding to 50% of soil water holding capacity) and this moisture content was maintained during the study. Several representative pots were weighed four times per week to estimate the water loss and pots were watered accordingly. The treatments included (a) control (CK), (b) (NH_4_)_2_SO_4_ (NF), (c) (NH_4_)_2_SO_4_ + straw (NS). The (NH_4_)_2_SO_4_ was added at rate of 250 kg N ha^-1^(0.1g N kg^-1^ soil),and Ca(H_2_PO_4_)_2_.H_2_O and K_2_SO_4_ were added as basal phosphorus and potassium fertilizer to all treatments at rates of 375 kg P_2_O_5_ ha^-1^ (0.15 g P_2_O_5_ kg^-1^ soil) and 250 kg K_2_O ha^-1^ (0.1 g K_2_O kg^-1^ soil). Rice straw, with a C to N ratio of 60 and sieved (< 2 mm) before use, was added at a rate corresponding to 1250 kg ha^-1^. On April 3, 2014, wheat (*Triticum aestivum* variety: Liaochun 18) was sown at a depth of 1 cm. After germination, eight uniform seedlings were selected and the remaining plants were uprooted. There were 16 containers for each treatment and 48 pots in total, which were distributed randomly on a flatbed in the wire house. Soil was sampled four times: at the seedling (April 18), jointing (May 13), filling (June 4) and maturity (July 8) stages. Soil samples were sieved (< 2 mm) and stored at 4°C. Four replicates per treatment were destructively sampled to determine soil pH, total organic C, total N, exchangeable NH_4_^+^, NO_3_^-^, microbial biomass N, microbial biomass C, soil extractable C, and activities of four enzymes, namely protease, β-D-glucosidase, N-acetyl-β-D-glucominidase, and cellobiohydrolase. Protease and N-acetyl-β-D-glucominidase activities are involved in the hydrolysis of protein and chitin while β-D-glucosidase and cellobiohydrolase are involved in cellulose degradation. Straw mainly consists of protein and cellulose, and because chitin is present in soils as fungal and macro faunal residue, we choose these four enzymes. All the other parameters were chosen for analysis because they are correlated with uptake of glycine by soil microorganisms. Soil mineral N was considered as the sum of soil NH_4_^+^ and NO_3_^-^. In each pot, the roots and aboveground parts of one whole plant were collected at seedling, jointing, filing and maturity stages. The roots were shaken with water for 30 min and any adhering soil was cleaned with a jet of distilled water. All plant samples were dried at 55°C until the weight was constant before being ground for analysis. Plant total N and biomass were also measured to show how plant growth and N content affect the uptake of amino acids by soil microorganisms.

### Analysis of soil properties

Soil pH was measured with a pH meter (FE20, Mettler Toledo, Switzerland) in a paste of 1:2.5 (w:v) of air-dried soil and deionized water. Soil and plant samples were analyzed for total organic C and total N by dry combustion (Carlo Erba CNS analyzer NA 1500 series 2). Exchangeable NH_4_^+^-N and NO_3_^-^-N were extracted from the field-moist samples using 2 M potassium chloride (KCl) and determined with a continuous flow analyzer (AA_3_, Bran + Luebbe, Germany). The automated procedure for the determination of NH_4_^+^-N was based on chlorination to monochloramine which reacts with salicylate to form 5-aminosalicylate. After oxidation and oxidative coupling the green color was measured at 660 nm. The NO_3_^-^-N was determined based on the hydrazinium reduction method: NO_3_^-^ was reduced to nitrite by hydrazinium sulphate and the nitrite reacted with sulphanilamide and a-naphthylethylnediamine dihydrochloride to form a highly colored azo dye. The color was measured at 540 nm.

Microbial biomass was determined by the chloroform fumigation extraction method [33]. Briefly, fresh soil (10 g) was incubated in the dark at room temperature whereas a complementary sample was fumigated with chloroform for 24 h. Both fumigated and non-fumigated samples were extracted with 40 mL 0.5 M K_2_SO_4_ on a reciprocal shaker for 30 min before filtering. The microbial biomass C was calculated as the difference in extractable C concentrations between the fumigated and unfumigated samples divided by a *K*_EC_ value of 0.45. The microbial biomass N was calculated as the difference in extractable N concentrations between the fumigated and unfumigated samples divided by a *K*_EN_ value of 0.54. The concentration of extractable C and extractable N were determined by a C and N analyzer (VarioTOC Analyzer, Elementar, Germany). The extractable C concentrations of unfumigated samples was considered to be the soil extractable C.

### Analysis of enzyme activities

Protease activity was determined as described by Ladd and Butler (1972) [26]. The activities of β-D-glucosidase, N-acetyl-β-D-glucominidase and cellobiohydrolase were assayed using fluorimetric substrates as described by Marx et al. (2001) [34]. The substrates of β-D-glucosidase, N-acetyl-β-D-glucominidase, and cellobiohydrolase were 4-methylumbelliferyl-β-D-glucopyranoside, 4-methylumbelliferyl -N-acetyl-β-D-glucominide and 4-methylumbelliferyl-β-D-cellbiopyranoside, respectively. The substrates were obtained from Sigma-Aldrich (St. Louis, USA). Briefly, substrates were dissolved in 1 ml of methyl cellosolve. Soil suspensions were prepared with 1:100 soil to 1mM NaN_3_ solution, and stirred for 15 min on a magnetic stir plate. Fluorimetric substrate solution (100μL) was combined with 50 μL soil homogenate and 50 μL 0.2 M acetate buffer (pH 5.0), pipetted into a 96-well microplate (8 wells per substrate), and incubated for 4 h at 30°C. The reaction was stopped by adding 50 μL 0.5 M NaOH, and the fluorescence determined immediately by a fluorometer (BMG Labtech, Offenburg, Germany) at 360 nm excitation and 460 nm emission. Each plate included a known standard of the product (4-methylumbelliferone; MUB), soil suspension controls and substrate controls. Soil suspensions controls contained substrate, buffer and NaN_3_, but no soil suspensions. Substrate controls were prepared by adding buffer to soil suspensions, but no substrate.

### Amino acid analysis

For the present study, ^13^C,^15^N-glycine was chosen because its metabolism has been well characterized, and it is a compound representing the fate of organic N sources [8, 11, 25]. In addition, glycine is one of the most abundant free amino acids in soil and can be utilized rapidly by microorganisms [35]. The concentration of ^13^C,^15^N-glycine in soil solution and the microbial biomass was measured using a compound-specific stable isotope analysis procedure described by Geisseler and Horwath (2014) [5]. Briefly, two samples were prepared for each pot. Field moist soil (equivalent to 6 g dry soil) from each pot was weighed into 40-ml glass vials and 0.37 mL of a solution containing ^13^C,^15^N-glycine dissolved in deionized water were added to the soil at a rate equivalent to 5 mg N kg^-1^ dry soil. With this addition the water content was raised to 60% of water holding capacity. The atom% ^15^N and ^13^C at the C_2_ position were 98% and 99% respectively. The treatment solutions were applied uniformly using a syringe with needle to ensure even distribution of the amino acid solution in the sample. The vials were then placed into a 12-L plastic container with a lid, lined with moist paper towels to minimize evaporation, and incubated at room temperature (20°C) for 4 h. One sample was extracted immediately after the predetermined incubation time, while the other was fumigated with chloroform for 24 h before extracting [33]. Geisseler and Horwath (2014) reported that amino transferases are released during fumigation, and this may affect the concentration of dual-labeled amino acids in the fumigated samples. In order to inactivate these enzymes, 0.1 mL 5% sodium dodecyl sulfate (SDS) was added to samples prior to fumigation. With this addition, the gravimetric soil moisture content was raised to 0.22 g water g^-1^ soil. Both fumigated and non-fumigated samples were extracted with 30 mL 0.5 M K_2_SO_4_ on a reciprocal shaker for 1 h, filtered, and the extracts were analyzed for ^13^C,^15^N-glycine.

In order to determine the optimal incubation time, a pre-experiment was conducted. Dual-labeled glycine (5 mg N kg^-1^) was evenly added to the untreated soil. The samples were then extracted immediately (at 0 h) or after 1, 2, 4, 6, 12, and 24 h. The extraction process and incubation method were as described above. The samples were then analyzed for ^13^C,^15^N-glycine in the soil solution and microbial biomass. Based on this experiment we chose an incubation time of 4 h for the main study.

Derivatization of nonvolatile dual-labeled glycine was performed in a fume hood prior to GC analysis using methyl chloroformate as by Smart et al. (2010) [36] and Geisseler and Horwath (2014) [5]. Briefly, 180 μL soil extract were placed in silanized borosilicate test tubes, and then 20 μL d-2,3,3,3-alanine solution (10 mg N L^-1^) were added as an internal standard. 167 μL methanol, 34 μL pyridine and 20 μL methyl chloroformate were added to samples, which were mixed vigorously with a vortex for exactly 30 s and then another 20 μL of methyl chloroformate were added. After mixing for 30 s, 400 μL chloroform were added, and mixed for 10 s. Finally, 400 mL of 50 mM NaHCO_3_ were added and samples were mixed for 10 s, upon which two layers separated. The aqueous (top) layer was removed with a glass Pasteur pipet. To ensure that no water was left in the organic (chloroform) layer, a few crystals of anhydrous Na_2_SO_4_ were added to dry the chloroform solution. The organic layer was then transferred into a 300-μL brown amber glass vial using a glass syringe.

The dual-labeled glycine derivatives were analyzed by a Thermo Scientific TRACE GC Ultra connected to a Thermo Scientific ITQ 1100 ion-trap mass spectrometer (Finnigan trace, Thermo Electron Co. Ltd., USA). The GC was equipped with an Agilent VF-1701ms capillary column (length 30 m, ID 0.25 mm, film thickness 0.25μm). The separation and analysis of methyl chloroformate derivatives were described by Smart et al. (2010). Briefly, samples (1 μL) were injected into the GC-MS under pulsed splitless mode (10 mL min^-1^ split flow, splitless time 1.0 min). Helium was used as carrier gas with the flow rate set at 1.0 mL min^-1^. The inlet temperature was 290°C. The GC oven temperature was increased from 50°C (2 min) to 180°C at a rate of 9°C min^-1^, to 220°C at 40°C min^-1^ and then at the same rate until up to 240°C (held for 11.5 min) and finally to 280°C at 40°C min^-1^ and held for 2 min. The interface temperature was set to 250°C and the ion trap temperature was 150°C. The electron energy was 70 eV. Mass spectra were obtained in full-scan mode (38–650 m/z) starting at 4 min. The ^13^C,^15^N-glycine was measured by the intensity of m/z 90 at a retention time of 11.89 min, while d-2,3,3,3-alanine was determined by the intensity of m/z 106 at a retention time of 11.46 min.

### Statistical anylyses

Thermo Xcalibur 2.1 SP1 software was used to integrate the area under the peaks. To quantify the concentration of the glycine in the samples, standard curves for glycine were prepared by analyzing 5 samples at different concentrations in the expected recovery range.

The data reported are the mean values of four replicates. The effect of different treatments and sampling time (four growth stages) was tested for all variables using repeated measures ANOVA. Sampling time was the with-subject effects. Significant differences among the treatments at each sampling time wereidentified by one-way ANOVA followed by the Tukey test, and determined at *P*<0.05. Before analysis of variance, data were checked for normal distribution by the Kolmogorov-Sminoff test. All statistical calculations were performed with SPSS Statistics 17.0 (SPSS Inc., Chicago, USA). Graphs were designed with Origin 9.0.

## Results

### Soil chemical properties

Soil pH was lower in the two treatments with N additions compared with the CK treatment throughout the entire wheat growth period (*P*<0.05) (Table 1). Repeated measures ANOVA showed significnat effects of sampling time and treatment, the within-subjects factor, on exchangeable NH_4_^+^-N NO_3_^-^-N (*P*<0.0001, repeated measures analysis). At the seedling stage, the soil exchangeable NH_4_^+^-N concentration was highest in the NF treatment, followed by the NS treatment and CK (Table 1). Thereafter, the exchangeable NH_4_^+^-N concentration decreased quickly in both NF and NS treatments. Throughout the experiment, significantly higher NO_3_^-^-N concentrations were observed in the treated soils (NF, NS) compared with CK. The highest NO_3_^-^-N concentrations occurred in the NF treatment and the addition of straw plus N fertilizer (NS) led to a significant decrease compared with the N addition treatment at all four sampling dates.

**Table 1:** Soil chemical properties (means± S.D., n = 4).

Stage	Treatment	pH	NH_4_^+^-N	NO_3_^-^-N
			mg·kg^-1^	
Seedling	CK	5.65(0.04)a	12.74(0.78)c	23.51(3.10) c
	NF	5.43(0.05)b	29.48(0.87)a	40.07(3.29)a
	NS	5.41(0.02)b	18.13(0.85)b	34.47(1.08)b
Jointing	CK	5.93(0.04)a	16.08(1.08)a	2.48 (0.62)c
	NF	5.48(0.06)c	15.14(0.44)a	22.06(3.58)a
	NS	5.64(0.06)b	13.01(0.86)b	6.50 (0.57)b
Filling	CK	6.17(0.14)a	22.37(0.14)a	1.83 (0.01)c
	NF	5.72(0.09)b	21.85(0.14)a	17.56(0.57)a
	NS	5.82(0.03)b	20.96(1.09)a	5.37 (0.37)b
Maturity	CK	6.31(0.20)a	15.70(1.16)a	1.87 (0.29)c
	NF	6.00(0.05)b	15.80(0.99)a	16.87(3.28)a
	NS	5.98(0.04)b	11.27(1.51)b	5.73(0.37)b

Different lowercase letters indicate significant differences between treatments at *P*<0.05.

The three treatments are: CK, control; NF, (NH_4_)_2_SO_4_; NS, (NH_4_)_2_SO_4_ + rice straw.

### Soil microbial biomass C, microbial biomass N and extractable C

**Fig 1:**
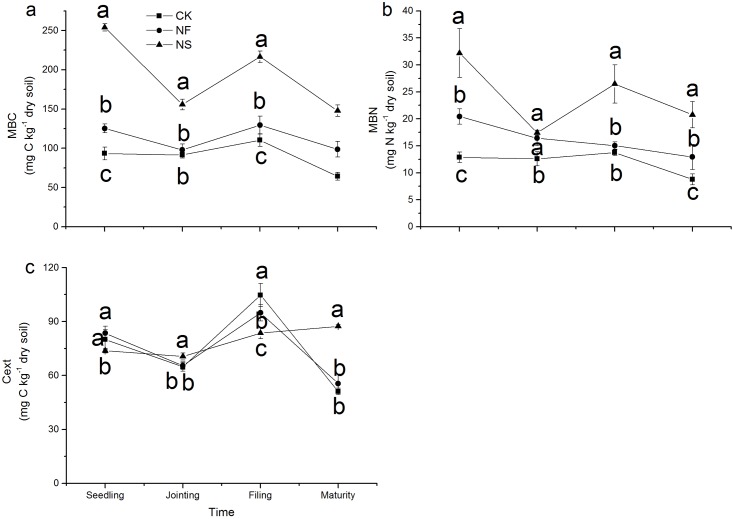
Changes in microbial biomass C (a), microbial biomass N (b), soil extractable C (c) in different treatments. Data points represent the mean of four replicates and error bars are the standard deviation of the mean. The three treatments are: CK, control; NF, (NH_4_)_2_SO_4_; NS, (NH_4_)_2_SO_4_ + rice straw. Different letters at each sampling date indicate a significant difference between different treatments at *P*< 0.05.

Both microbial biomass C and microbial biomass Nwere significantly increased by the straw addition throughout the experiment, following a similar trend over time (Fig 1a and 1b). In the NS treatment, the highest microbial biomass C and N were observed at the seedling stage; thereafter, they gradually decreased until the end of experiment. In the NF and control treatments, the changes in microbial biomass C over time were less pronounced. The microbial biomass Ngradually decreased with sampling stage in the NF treatment, andwas higher than that in CK.

The changes in extractable C concentrations over time were similar to those observed for microbial biomass C, except in the NS treatment, where extractable C increased between filling stage and maturity (Fig 1c).

### Soil enzyme acitivities

**Fig 2:**
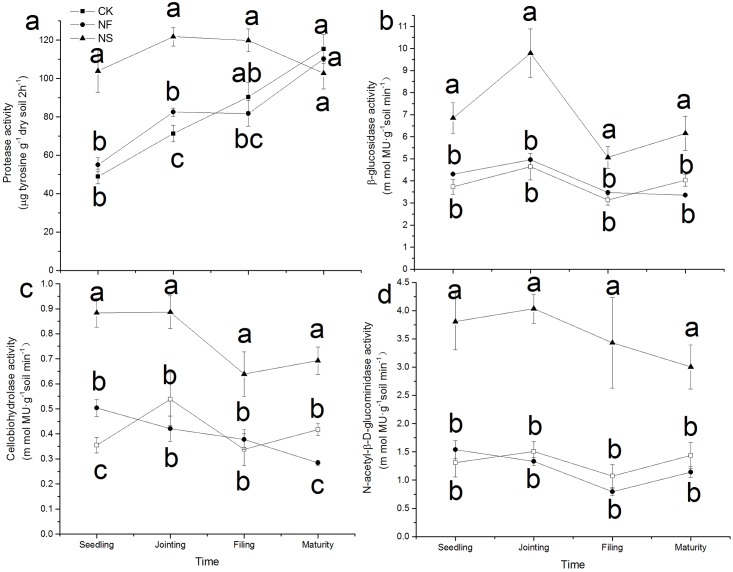
Potential enzyme activities as affected by different treatments at 4 sampling dates in the pot experiment. Data points represent the mean of four replicates and error bars are the standard deviation of the mean. The three treatments are: CK, control; NF, (NH_4_)_2_SO_4_; NS, (NH_4_)_2_SO_4_ + rice straw. Different letters at each sampling date indicate a significant difference between different treatments at *P* < 0.05.

The addition of straw (NS treatment) significantly increased soil protease activity during the first three sampling dates (Fig 2a), compared to the other treatments. The addition of N alone (NF treatment) significantly increased protease activity compared with CK at the jointing stage, while no significant differences were observed at the other three sampling stages. In both CK and NF treatments, protease activity increased over time. The application of straw plus N fertilizer (NS treatment) also significantly increased β-D-glucosidase, cellobiohydrolase, and N-acetyl-β-D-glucominidase activities throughout the entire growth period compared with CK and NFtreatments (Fig 2b, 2c and 2d). The N fertilizer alone (NF) did not changeβ-D-glucosidase, cellobiohydrolase, and N-acetyl-β-D-glucominidase activities compared with CK during the whole growth season.

### Amino acid turnover

**Fig 3:**
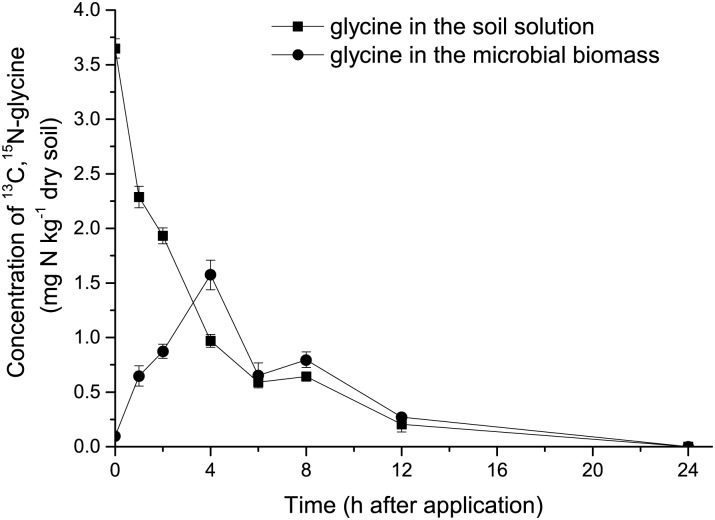
The concentration of the added ^13^C,^15^N-glycine in the control soil solution and in the microbial biomass at different incubation times.

When soil samples were extracted immediately after ^13^C,^15^N-glycine additions, 75% of the added glycine was recovered from the solution (Fig 3). Microbial utilization of dual-labeled glycine proceeded rapidly. The concentration of added glycine in soil solution decreased linearly during the initial 4 h from 3.65 to 0.97 mg N kg^-1^ soil. After 12 h, the amount of dual lableled glycine in soil solution corresponded to only 4% of the glycine added, and it was no longer detectable in solution after 24 h. The maximum concentration of added glycine in the microbial biomass was reached after 4 h, which corresponds to 31% of the added amount. Labeled glycine was no longer detectable in the microbial biomass after 24 h.

**Fig 4:**
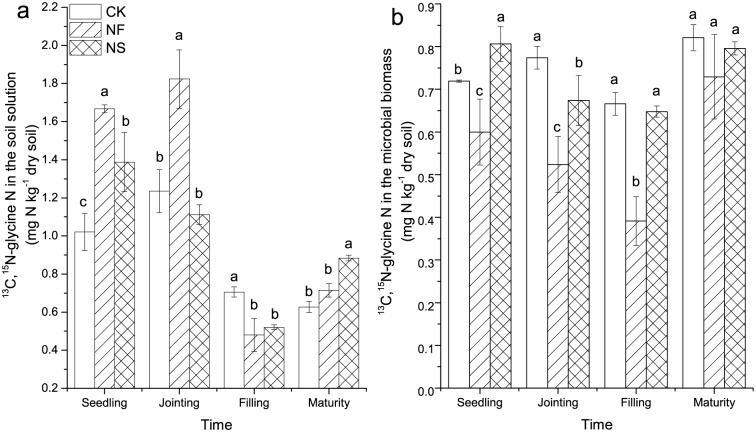
The amount of added ^13^C,^15^N-glycine in soil solution and in the microbial biomass under different treatments and different sampling times. Data points represent the mean of four replicates at each sampling time and error bars are the standard deviation of the mean. Different lowercase letters indicate significant differences between treatments at *P*<0.05. Different capital letters indicate significant differences among sampling times for the same treatment at *P*<0.05. The three treatments are: CK, control; NF, (NH_4_)_2_SO_4_; NS, (NH_4_)_2_SO_4_ + rice straw.

**Fig 5:**
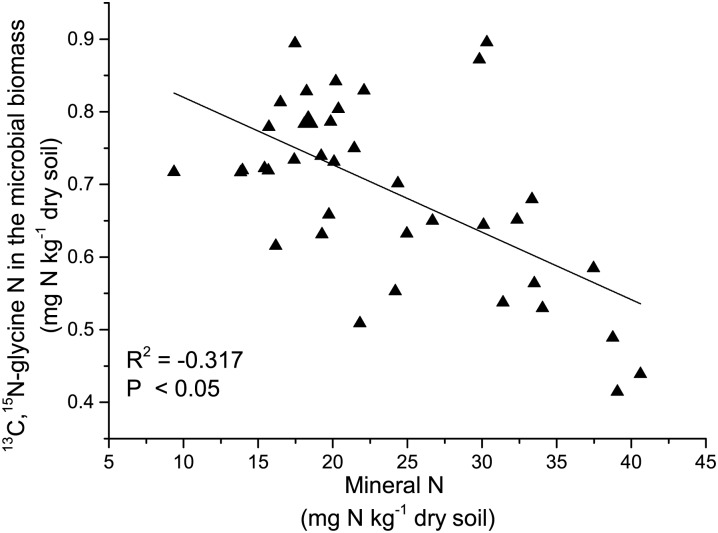
Correlation between ^13^C,^15^N-glycine in the microbial biomass and soil mineral N.

Dual—labeled glycine in the soil solution and in the microbial biomass differed significnantly among treatment on four growth stages (*P* < 0.0001, repeated measures ANOVA). When extracted 4 h after addition, a larger proportion of the added glycine was detected in soil solution during vegetative growth (seedling and jointing) than during reproductive growth (filling and maturity) (Fig 4a). During vegetative growth of wheat, ^13^C,^15^N-glycine in soil solution was the highest in the NF treatment. The N fertilizer addition alone (NF treatment) significantly decreased assimilation of intact glycine by soil microorganisms compared with CK, but it increased the glycine uptake compared to the NF treatment on the first three sampling dates, and all treatments were no longer different at harvest (Fig 4b). The relative amount of uptake of ^13^C,^15^N-glycine by soil microorganisms decreased from the seedling to the filling stage, but increased at the maturity stage. In addition, uptake of ^13^C,^15^N-glycine in the microbial biomass was negatively correlated with mineral N (Fig 5).

### Total wheat biomass and the total N content

Application of N fertilizer with and without straw significantly enhanced the plantbiomass relative to control throughout the experiment (Table 2), while no significant difference was observed between the NF and NS treatments.

**Table 2:** Total wheat biomass and the Total N content at four sampling times under pot conditions (means ± S.D., n = 4).

Stage	Treatment	Total biomass	Shoot TN	Root TN
		g pot^-1^	g·kg^-1^	
Seedling	CK	0.32(0.014)b	61.23(0.71)b	29.52(0.18)a
	NF	0.37(0.030)a	66.42(2.32)a	29.77(0.24)a
	NS	0.42(0.043)a	62.81(2.16)a	28.80(0.24)a
Jointing	CK	3.44(0.35)b	31.92(1.71)c	7.86(0.52)c
	NF	4.65(0.37)a	51.44(3.72)a	15.05(0.56)a
	NS	4.54(0.48)a	44.46(4.23)b	11.45(0.61)b
Filling	CK	7.80(0.48)b	10.21(0.84)c	9.80(0.28)c
	NF	9.73(0.66)a	22.93(0.86)a	15.74(1.61)a
	NS	10.29(0.23)a	15.34(1.71)b	11.58(0.70)b
Maturity	CK	10.39(0.56)b	7.96(0.73)c	10.21(0.19)a
	NF	12.68(1.43)a	14.01(0.85)a	10.57(0.11)a
	NS	13.44(0.69)a	10.32(1.56)b	10.72(1.06)a

Different lowercase letters indicate significant differences between treatments at *P*<0.05.

The three treatments are: CK, control; NF, (NH_4_)_2_SO_4_; NS, (NH_4_)_2_SO_4_ + rice straw.

At the seedling stage, the total N content of shoots and roots was highest compared to the other sampling stages, and both total N contents decreased with wheat growth (Table 2). A significant treatment effect on shoot total N was found at the latter three growth stages with the highest content in the NF treatment and the lowest in the control. The total N content of roots showed the same pattern as that of shoot total N content at the jointing and filling stages of wheat growth.

## Discussion

Using a combination of compound-specific stable isotope analysis and chloroform fumigation-extraction provided direct evidence for the uptake of intact amino acids by soil microorganisms in this pot experiment. Application of N fertilizer and straw affects the dynamics of amino acids and microbial biomass during the wheat growing season. Following its addition, ^13^C,^15^N-glycine in soil solution decreased rapidly and was not detected after 24h (Fig 3). The half-life of glycine was 3.0 h which is consistent with Jones et al. (2009) [37], who reported a half-life of amino acids ranging from 0.8 to 5.9 h in different soils. Microorganisms may assimilate amino acids to meet their need for C, N, and energy [5, 38]. Glycine uptake was influenced by the application of N and straw (Fig 4). In the NF treatment, the uptake of ^13^C,^15^N-glycine by soil microorganisms decreased throughout the wheat growing season. And the amount of ^13^C,^15^N-glycine found in the microbial biomass was negatively correlated with mineral N, which indicates that inorganic N was the preferred N source for soil microorganisms when the availability of mineral N was higher. Previous research has shown that inorganic N is the preferred source of N for bacteria and fungi and that enzyme systems responsible for the utilization of alternative N sources, such as amino acids, are repressed when inorganic N concentrations are high [5, 22, 38–40]. In NF treatment, the ratio of amino acid N with total N decreased because N fertilizer application increased soil total N (S1 Fig), which likely contributed to the decreased assimilation of ^13^C-^15^N glycine by soil microorganisms. Furthermore, the addition of N fertilizer alone caused only a small increase in microbial biomass N and microbial biomass C in the current research, which suggests that the demand for N and C did not change drastically (Fig 1a and 1b), and that the demand for N was likely met by soil mineral N. Under these conditions, uptake of amino acids by soil microorganisms as an N source may decline [25].

The decomposition of straw in soil exerted a strong effect on the utilization of glycine by soil microorganism despite the fact that N was added at the same rates in both the NF and NS treatments. Protease, β-D-glucosidase and cellobiohydrolase, and N-acetyl-β-D-glucominidase are involved in the hydrolysis of protein, cellulose, and chitin, respectively [28, 41, 42]. Increase in the activity of these enzymes makes more C available to soil microorganisms [24], which can be confirmed by significant increase in microbial biomass C and microbial biomass N (Fig 1a and 1b). Furthermore, the presence of rice straw also implies an additional addition of microorganisms to soil and this was reflected in the strong increase in soil microbial C and microbial N. Under such conditions, a large amount of N was needed. This was demonstrated by the lower exchangeable soil inorganic N in NS treatment (Table 1), indicating the occurance of net N immobilization. Amino acids may meet the microbialneed for extra N under such circumstances [43]. As expected, microbial uptake of ^13^C,^15^N-glycine was thereforehighest in the NS treatment. In the control treatment, the mineral N concentration was the lowest because no extra N was added in this treatment, and amino acids were the primary source of N available to soil microorganism to meet their N requirements; this may explain the higher propensity for ^13^C,^15^N-glycine uptake by soil microorganisms in the control compared to the NF treatment.

Plant residue pools consist of three components based on the rates of decomposition: rapidly (carbohydrates or amino acids), intermediately (cellulose and hemicellulose), and slowly decomposing components (lignin-like compounds), [44]. The soluble component of residues, including amino acids, which decomposes rapidly in the initial stage, may be readily absorbed by soil microorganisms to meet growth requirements. Therefore, the decomposition of plant residues may promote the uptake of amino acids by soil microorganisms, which was observed as higher uptake of an addition of^13^C-^15^N glycine at the seedling stage. Thereafter, the decomposition of the straw was slow and the growth of wheat relatively rapid, leading to strong competition for N between the plant and microorganisms. For this reason the amount of the^13^C,^15^N-glycine addition which was absorbed by soil microorganisms was lower at the jointing and filling stages.

Total N content in wheat biomass showed different trends between the NF and NS treatments. The addition of N fertilizer alone increasedtotal N content of wheat compared with the NS treatment, while soil microbial biomass C and microbial biomass N in the NF treatment were lower than in the NS treatment, probably because microorganisms competed poorly with wheat for N in the NF treatment. The ^13^C,^15^N-glycine assimilated by soil microorganisms was also lower in the NF treatment. We did not measure glycine uptake by wheat roots,although it is known that wheat (*Triticum aestivum*) can take up glycine from soil [45, 46]. Total N content of wheat in the NS treatment decreased significantly compared with that of the NF treatment. A large amount of N, including dual-labelled glycine, was reserved in soil microbial biomass,probably explaining the marked increase in soil microbial biomass C and microbial biomass N in the NS treatment. In addition to N, C is also required as the fundamental building block of biomass and an energy source during the proliferation of soil microorganisms[5]. An optimal balance between C and N supply can maintain high microbial activity in soil.

## Conclusions

The addition of nitrogen fertilizer with or without straw can affect the dynamics of microbial amino acid uptake and microbial biomass as a whole during the wheat growing season. Uptake of intact glycine was low in the NF treatment, indicating that mineral N is the major N source for microbial growth. Enzyme activities as well as microbial biomass C and microbial biomass N increased by adding straw. Soil inorganic N decreased simultaneously and plant N also decreased markedly, indicating strong consumption of N by soil microorganisms. With straw application, uptake of intact ^13^C,^15^N-glycine by soil microorganisms increased, showing that amino acids can supply additional N when inorganic N is exhausted. Based on these observations, we can confirm that substrate availability affects microbial uptake of glycine, which also simultaneously affects N uptake by plants.

## Supporting Information

S1 Fig The percentage of double glycine and soil total nitrogen under different treatments and different sampling times. Data points represent the mean of four replicates at each sampling time and error bars are the standard deviation of the mean. Different lowercase letters indicate significant differences between treatments at *P*<0.05. The three treatments are: CK, control; NF, (NH_4_)_2_SO_4_; NS, (NH_4_)_2_SO_4_ + rice straw.
(TIF)
